# MOG encephalomyelitis: distinct clinical, MRI and CSF features in patients with longitudinal extensive transverse myelitis as first clinical presentation

**DOI:** 10.1007/s00415-020-09755-x

**Published:** 2020-02-13

**Authors:** Julia Loos, Steffen Pfeuffer, Katrin Pape, Tobias Ruck, Felix Luessi, Annette Spreer, Frauke Zipp, Sven G. Meuth, Stefan Bittner

**Affiliations:** 1 Department of Neurology, Focus Program Translational Neuroscience (FTN), Rhine Main Neuroscience Network (rmn2), University Medical Center of the Johannes Gutenberg-University of Mainz, Mainz, Germany; 2 Clinic of Neurology with Institute of Translational Neurology, University of Muenster, Muenster, Germany

**Keywords:** Myelin oligodendrocyte glycoprotein (MOG) antibodies, Diagnosis, Myelitis, Longitudinal extensive transverse myelitis (LETM), Neuromyelitis optica spectrum disorders (NMOSD)

## Abstract

**Background:**

Based on clinical, immunological and histopathological evidence, MOG-IgG-associated encephalomyelitis (MOG-EM) has emerged as a distinct disease entity different from multiple sclerosis (MS) and aquaporin-4-antibody-positive neuromyelitis optica spectrum disorder (NMOSD). MOG-EM is associated with a broader clinical phenotype including optic neuritis, myelitis, brainstem lesions and acute disseminated encephalomyelitis with a substantial clinical and radiological overlap to other demyelinating CNS disorders.

**Objective:**

To evaluate common clinical, MRI and CSF findings, as well as therapy responses in patients with longitudinal extensive transverse myelitis (LETM) as initial clinical presentation of MOG-EM.

**Methods:**

After excluding patients with a known diagnosis of MS, we identified 153 patients with myelitis of which 7 fulfilled the inclusion criteria and were investigated for MRI, CSF and clinical parameters.

**Results:**

Patients with LETM as first clinical presentation of MOG-EM display similar characteristics, namely a lack of gadolinium-enhancement in spinal cord MRI, marked pleocytosis, negative oligoclonal bands, a previous history of infections/vaccinations and response to antibody-depleting treatments for acute attacks and long-term treatment.

**Conclusions:**

We identify common pathological findings in patients with LETM as first clinical presentation of MOG-EM which distinguishes it from other forms of LETM and should lead to testing for MOG-IgG in these cases.

## Introduction

Myelin oligodendrocyte glycoprotein (MOG) is an encephalitogenic protein on CNS oligodendrocytes that can initiate demyelinating autoimmune responses in experimental models of inflammatory demyelinating diseases [1]. After years of conflicting research on the role of MOG-IgG antibodies in neuroinflammatory diseases, the methodological improvements using more reliable cell-based assays presenting human MOG protein in a biosimilar conformation have identified typical clinical presentations associated with anti-MOG-IgG antibodies, now called MOG-IgG-associated encephalomyelitis (MOG-EM). In the past few years, growing clinical, immunological and histopathological evidence suggests that MOG-EM can now be considered as a clearly distinct disease entity from both multiple sclerosis (MS) and aquaporin-4-positive neuromyelitis optica spectrum disorder (NMOSD) [2–5]. Terms like “MOG-positive NMOSD” should, therefore, be avoided to clearly separate MOG-EM, which has a different antibody-mediated pathology and broader clinical spectrum, from aquaporin-4 associated NMOSD [1, 3]. While anti-myelin oligodendrocyte glycoprotein antibodies (MOG-IgG) were originally linked especially to acute disseminated encephalomyelitis (ADEM)-like presentation in pediatric patients [6, 7], more recent studies proposed a wider clinical spectrum including uni- and bilateral optic neuritis (ON), short and longitudinal extensive transverse myelitis (LETM), brainstem and cerebellar lesions or seizures [8–15].

Recently, for the first time, diagnostic criteria for MOG-EM have been proposed based on a combination of (1) seropositivity for MOG-IgG with (2) one of the clinical presentations described above and (3) MRI or electrophysiological findings compatible with CNS demyelination [2]. Due to a substantial overlap in clinical and radiological presentations between MOG-EM and other acquired demyelinating CNS disorders, deciding which clinical and paraclinical findings should be accompanied by MOG-IgG testing, especially at first clinical presentation, remains an evolving challenge. For example, isolated uni- or bilateral optic neuritis has been reported as the most common symptom at MOG-EM disease onset (64% and 73% of patients in a German and an Australasian/New Zealand cohort of patients with MOG-EM) [8, 16] and isolated LETM was the first clinical symptom in only 11% and 18% of these patients. This lies in the range of 7.4–23.2% of previously reported prevalence of MOG-IgG seropositivity in AQP4-IgG-seronegative LETMs [4]. In these cohorts, patients with LETM as first clinical symptom were associated with a larger percentage of residual disability, suggesting that early and aggressive treatment would be particularly warranted in this patient population [16]. Apart from MOG-EM and NMOSD, LETM occurs in various other autoimmune inflammatory diseases such as neurosarcoidosis or Sjögren syndrome [17]. So far, details about clinical, MRI and radiological findings of patients with LETM as a first clinical manifestation of MOG-EM are still rare, especially in cohorts exclusively including adults. In our study, we investigated characteristics of such patients in a cohort of seven individuals presenting with isolated MOG-IgG-positive LETM, identified from a larger cohort of 62 patients with LETM. We report cerebrospinal fluid (CSF) and magnetic resonance imaging (MRI) data, clinical presentation and disease development under B cell-depleting therapy. We present common characteristics of MOG-EM patients with LETM at initial presentation, which will help distinguish this condition from alternative inflammatory-mediated spinal cord affections.

## Methods

### Patients

**Fig. 1:**
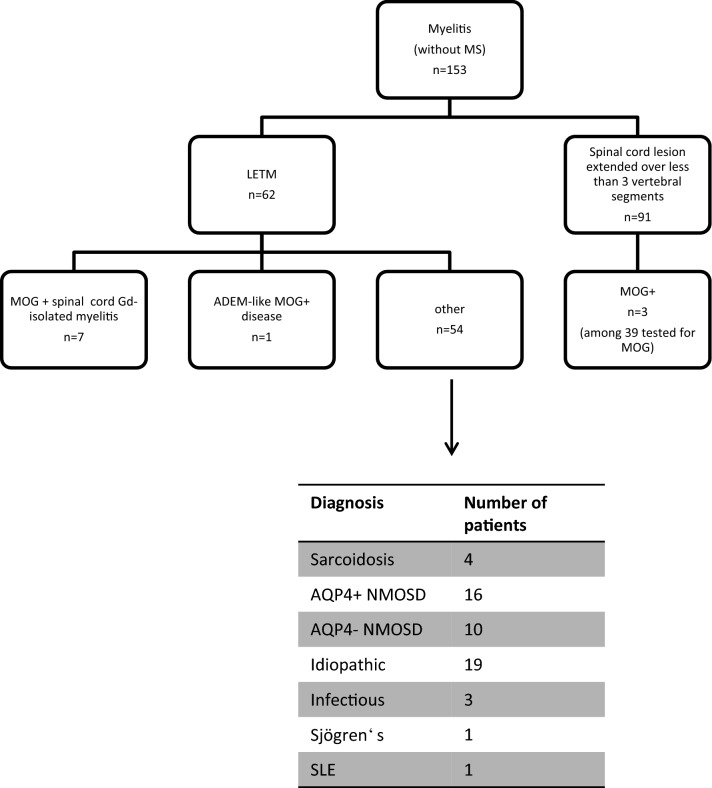
Flow chart demonstrating selection of patients for further analysis and distribution of etiology of LETM in patients. *ADEM* acute disseminated encephalomyelitis, *AQP4* aquaporin 4, *LETM* longitudinal extensive transverse myelitis, *MOG* myelin oligodendrocyte glycoprotein, *MS* multiple sclerosis, *NMOSD* neuromyelitis optica spectrum disorder

Clinical and paraclinical data of patients who presented with myelitis between 2010 and 2018 in the university medical centers of Mainz and Münster were evaluated. Data were obtained for routine clinical assessment and retrospectively evaluated. Patients who had been diagnosed with MS according to the revised McDonald criteria [18] were excluded from the study. We identified 153 myelitis patients of which 91 showed spinal cord lesions extended over less than three vertebral segments and were excluded from further analysis. In this subgroup, 39 patients were tested for MOG-IgG antibodies and 3 tested positive. 62 patients showed longitudinal extensive transverse myelitis (LETM) defined as at least one spinal cord lesion extending over more than three vertebral segments. In this cohort, eight patients tested positive for MOG-IgG antibodies in serum. Patients who did not show isolated LETM as first clinical presentation of MOG-EM were excluded from this study. One of the patients showed a combination of LETM with severe cerebral ADEM and more than 15 cerebral gadolinium enhancing lesions and was, therefore, excluded. In total, seven patients presented with isolated myelitis and were further evaluated (see Fig. 1). Patients were followed up for an average of 31 months after disease initiation (range 6–68 months). Available brain and spinal cord MRI data were evaluated and included axial and sagittal images of the brain and spinal cord obtained by T1-weighted, T2-weighted and fluid-attenuated inversion recovery (FLAIR). MRI data were obtained during primary manifestation of disease before start of treatment. See Table 1 for all patient data.

**Table 1:** Overview of clinical findings of individual patients

	Summary	Patient 1	Patient 2	Patient 3	Patient 4	Patient 5	Patient 6	Patient 7
Sex (m/f)	3/4	Male	Male	Female	Female	Female	Female	Male
Age, years (median + range)	31.7 (21–41)	24	37	21	41	26	34	39
Infections/ vaccination prior to disease	5/7	Skin infection left foot	GI infection, skin efflorescences	Viral infection of respriratory tract	Vaccination	Unclear	Pyelonephritis	Unclear
Cranial MRI	No abnormal findings	No abnormal findings	No abnormal findings	No abnormal findings	No abnormal findings	No abnormal findings	No abnormal findings	No abnormal findings
Spinal Cord MRI	LETM	LETM: whole spine	LETM: thoracic spine	LETM: Th3-10	LETM: C2-6	LETM: Medulla oblongata-C9	LETM: C2-6	LETM: Th2-11
Gadolinium-uptake in sMRI	Negative	Negative	Negative	Negative	Negative	Negative	Negative	Negative
Optic neuritis	Negative	Negative	Negative	Negative	Negative	Negative	Negative	Negative
CSF cells (mean + range)	196 (49–353)/µl	136/µl	325/µl	264/µl	179/µl	49/µl	353/µl	67/µl
Neutrophil granulocytes in CSF	2/7 positive	No	No	Yes	No	No	Yes	No
CSF protein (mean + range)	464,7 (64 – 1100) mg/dl	141 mg/dl	64 mg/dl	77 mg/dl	784 mg/dl	150 mg/dl	1100 mg/dl	937 mg/dl
OCB	Negative	Negative	Negative	Negative	Negative	Negative	Negative	Negative
CSF Lactate (mmol/l)	2.5 (mean)	2.5	3.1	2.9	1.8	2.1	3.4	1.7
QAlb	13.61 (mean)	17.65	9.25	14.52	11.36	18.49	10.58	13.39
CSF IgA (mg/l)	11.57 (mean)	25.3	7.68	8.73	14.74	4.93	12.11	7.51
CSF IgG (mg/l)	104 (mean)	159	56	72	172	93	72	104
CSF IgM (mg/l)	6.27 (mean)	18.8	1.2	15.3	1.8	2.4	1.1	3.3
MRZ reaction	Negative	positive for VZV (1.95)	Not tested	Not tested	Positive for VZV (1.8)	Neg	Neg	Positive for measles (2.1)
MOG-Ab in serum (range)	1:32–1:3200	1:320	1:32	1:100	1:32	1:100	1:3200	1:100
MOG-AB status in remission	5/7 negative	Negative	Negative	Not tested	Positive	Negative	Negative	Negative
AQP4 antibodies	Negative	Negative	Negative	Negative	Negative	Negative	Negative	Negative
Second relapse	1/7	No	No	No	Yes	No	No	No
Acute therapy	Recovery on IVMPS, IVIG or plasma exchange	Recovery on IVIG	Recovery on plasma exchange	Recovery on plasma exchange	Recovery on plasma exchange	Recovery on plasma exchange	Recovery on plasma exchange	Partial recovery on IVMPS
Immunotherapy	Induction with rituximab (six patients) or cyclophosphamide(1 patient)	Rituximab	Rituximab	Rituximab	Rituximab	Rituximab	Induction rituximabdeescalation to azathioprine	Induction of cyclophosphamide deescalation to azathioprine
Outcome	Marked recovery in four patients, partial recovery in three patients	Marked recovery	Marked recovery	Marked recovery	Partial recovery	Marked recovery	Partial recovery	Partial recovery

*CSF* cerebrospinal fluid, *MRI* magnetic resonance imaging, *IVIG* intravenous immunoglobulins, *IVMPS* intravenous methylprednisolone, *LETM* longitudinal extensive transverse myelitis, *MOG* myelin oligodendrocyte glycoprotein, *OCB* oligoclonal bands, *qAlb* albumin quotient, *AQP4* aquaporin 4, *VZV* varicella zoster virus

### MOG antibody detection

Anti-MOG-IgG antibody titers in serum and CSF were determined using cell-based assays (Anti-Myelin Oligodendrocyte Glycoprotein (MOG) IIFTT, EUROIMMUN AG) with native MOG as a substrate. Data on Anti-MOG-IgG antibody titers were obtained during routine clinical assessment of patients and retrospectively evaluated.

### Standard protocol approvals, registrations and patient consents

**Fig. 2:**
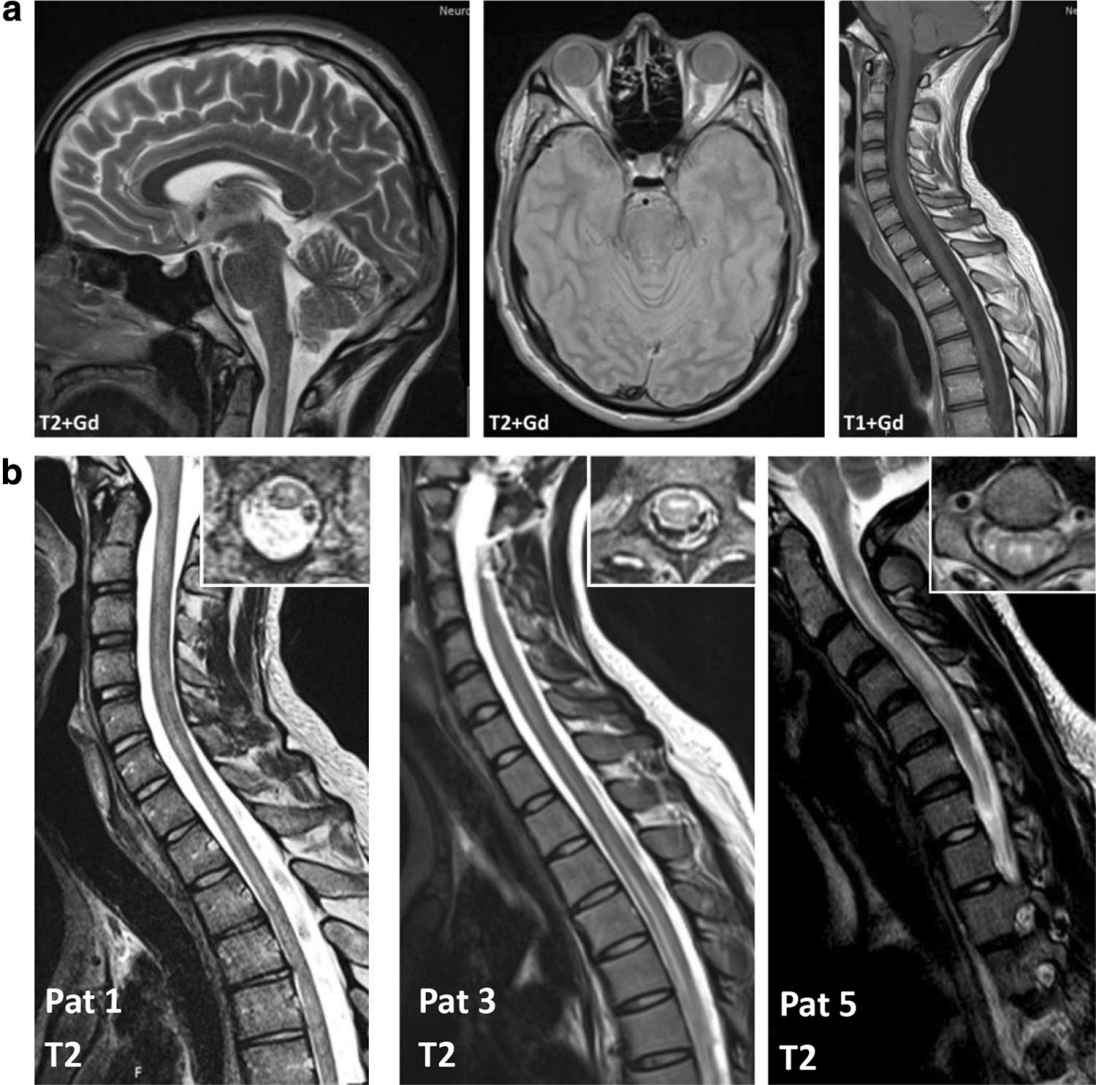
**a**Representative examples of brain MRI scans from one patient during peak of disease symptoms. No lesions were detected in either T2- or T1-weighted MRI images with gadolinium (Gd). **b** Representative examples of spinal cord lesions detected by MRI in each patient. Sagittal T2-weighted spinal MRI performed at disease onset show longitudinal lesions extending throughout the spinal cord. Inserts show axial sections of the spinal cord at lesion level

This study was approved by the local ethical committees and performed according to the Helsinki Declaration. All patients provided written informed consent (Fig. 2).

## Results

### Epidemiological data and clinical presentation

Of 153 patients who presented with myelitis in two expert centers, 62 showed longitudinal extensive transverse myelitis (LETM). In this cohort, eight patients tested positive for MOG-IgG antibodies in serum. In total, seven patients presented with isolated myelitis and were further evaluated (see Table 1). Patients were followed-up for an average of 31 months after disease initiation (range 6–68 months). Of the seven patients between 21 and 41 years (average 31.7 years), three were males and four females. This is in accordance with a generally higher rate of male patients with MOG-EM compared to NMOSD (only around 10–15%) [4]. Two patients had hypothyroidism, one had scoliosis and the other patients did not have any chronic disease. Four patients had acute infections prior to onset of myelitis symptoms including infections of the skin, gastrointestinal tract, respiratory system and acute pyelonephritis. One patient had received vaccination for diphtheria, pertussis, poliomyelitis and tetanus 2 weeks prior to disease onset. Of note, all patients presented with similar, rapidly developing symptoms of severe acute myelitis including paraplegia, sensory loss and urinary retention (mean EDSS 7.6, range 6.5–9).

### Patients with MOG-antibody positive LETM lack gadolinium enhancement in spinal cord MRI

Spinal cord lesions indicative of acquired demyelinating disease were detected in T2-weighted MRI images during the peak of disease symptoms in all patients. Lesions were mainly located in the cervical (3 out of 7 patients) or thoracic (3 out of 7 patients) spine. One patient showed lesions in the whole spine. On average, lesions extended over nine spinal segments (range 4–19 segments). We did not observe swelling or signs of necrosis of the spinal cord in any patients. In accordance with previous reports [13], lesions were located ventrally in the spinal cord with axial sequences showing T2 hyperintensity most prominent around the central canal. While gadolinium enhancement of spinal lesions is frequent in patients with NMOSD [19, 20], we could not detect gadolinium enhancing lesions at any time point in our patient cohort. Cranial MRI showed no abnormal findings in these patients at onset of disease. In six patients, information on follow-up cranial MRI was available. While five patients still showed negative cranial MRIs in the follow-up, one patient showed one small fronto-temporal white matter lesion without gadolinium enhancement that was evaluated as non-MOG-EM specific 6 years after initial manifestation of MOG-EM.

### Optic neuritis is not associated with episodes of isolated LETM as first clinical presentation of MOG-EM

Optic neuritis is a common feature in patients diagnosed with NMO or NMOSD. Moreover, both diseases are frequently associated with LETM [21, 22], which is also reflected in our cohort of LETM patients in which 26 out of 54 (48.1%) patients with LETM were positive for aquaporin-4-antibodies or met the 2015 Wingerchuk criteria [21]. Interestingly, in our patient cohort presenting with MOG-antibody positive LETM, none of the patients had clinically apparent optic neuritis during the first phase of their disease. Only one patient showed delayed P100 latencies in both eyes suggesting a subclinical optic nerve inflammation, but had not experienced an episode of optic neuritis. Visual evoked potentials (VEP) from other six patients were without abnormalities.

### MOG antibodies can be detected in serum during acute disease and can disappear in remission

All patients tested positive for MOG-IgG antibody in serum during the first clinical episode with titers ranging from 1:32 to 1:3200 (mean 1:555). No MOG antibodies could be detected in the CSF. This is in line with previous studies that showed that MOG-IgG is produced mainly in the periphery [23]. Remarkably, at remission, MOG antibodies could no longer be detected in five out of seven patients. All patients tested negative for Aquaporin-4 antibodies.

### Patients show lymphocytic pleocytosis and high protein levels in CSF, but no oligoclonal bands or MRZ reaction

Lumbar puncture and CSF collection was performed on all seven patients at disease onset. CSF-restricted oligoclonal IgG bands, indicative of intrathecal IgG synthesis, were not detected for any of the patients. All patients showed a type 1 pattern in the isoelectric focusing [24]. We could not detect mirror patterns in CSF analysis of our patient cohort. Moreover, MRZ reaction was negative in all patients. Protein levels in CSF ranged from 64 to 1100 mg/dl (mean 465 mg/dl). CSF/serum albumin quotient (Qalb) ranged from 9.25 to 18.49 (mean 13.61). CSF lactate was elevated in 5/7 patients (71.4%). In a previous study 42.9% of Aquaporin-4-positive NMO patients showed an elevation of CSF lactate levels [25]. All patients showed remarkable lymphocytic pleocytosis (mean 218 cells/µl, range 49–353 cells/µl, normal range: < 5 cells/µl).

### Patients respond well to plasma exchange and long-term B-cell depleting therapy

**Fig. 3:**
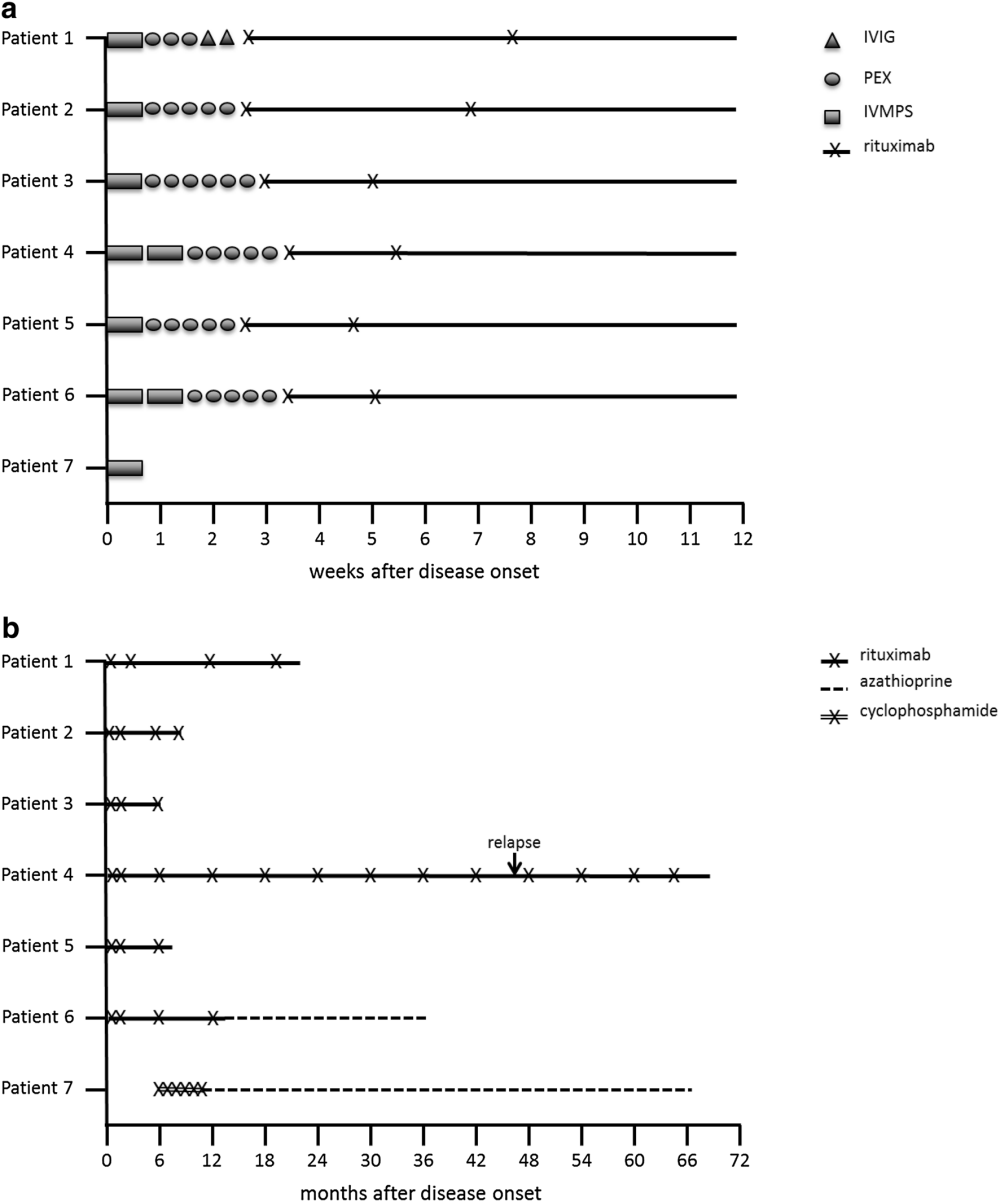
**a** Figure showing individual treatment regimen of patients (*y*-axis) during the first 12 weeks after disease onset (*x*-axis). Acute treatment is shown by different symbols (*IVIG* intravenous immunoglobulins, *PEX* plasma exchange, *IVMPS* intravenous methylprednisolone). Induction of long-term treatment is shown by lines, administrations are marked with an x. Patient seven did not receive long-term treatment during the first 12 weeks of disease. **b** Figure showing length and treatment regimen of long-term treatment. Administrations of rituximab/cyclophosphamide are marked with an x. Relapses are marked with an arrow

All individual disease courses are depicted in Fig. 3. Patients in the acute phase of MOG-EM were treated with glucocorticoids, plasma exchange and intravenous immunoglobulin therapy. In one case, a patient received only glucocorticoid treatment and only made a partial recovery (improvement from EDSS 7 to EDSS 5). The other six patients did not respond to glucocorticoids and received plasma exchange which was evaluated as effective in five cases. (EDSS decreased by at least 1). In one case only application of intravenous immunoglobulin showed efficacy.

After recovery from the initial episode of the disease, long-term immunotherapy was established in all patients. Six out of seven patients were treated with rituximab, five of whom did not have any relapses during follow-up (average observation period under rituximab treatment: 25 months). One of these patients was switched to azathioprine after 14 months of treatment. One patient suffered from an end-of-dose relapse after 45 months with a count of 30 CD19^+^ B-cells/µl at time of relapse. One patient was initially treated with cyclophosphamide, switched to azathioprine after 12 months and did not have any relapses during the 68 month observation period.

### LETM as first clinical presentation of MOG-EM is associated with positive clinical outcome

In our study four patients showed total recovery (EDSS 0) and three patients showed significant recovery but had residual symptoms including hypoesthesia, impaired bladder function and gait (EDSS range 1–6, mean EDSS 4). CSF protein levels in patients who recovered completely ranged from 49–150 mg/dl (mean 101 mg/dl), and were significantly higher (*p* < 0.05, Student’s *t* test) in patients with residual symptoms after treatment (mean 940 mg/dl, range 784–1100 mg/dl). Moreover, we observed higher MOG antibody titers (range 1:32–1:320) in patients who recovered completely than in patients who did not (range 1:10–1:100). Lymphocytic cell count in the CSF also differed between the two groups. Patients who recovered completely showed high lymphocytic cell counts (mean 270 cells/µl, range 136–353 cells/µl), whereas lymphocytic cell count in patients who did not recover completely were still elevated but significantly lower [mean 98 cells/µl, range 49–179 cells/µl; (*p* < 0.05, Student’s *t* test)]. Initial clinical severity of disease did not have an influence on the outcome as there was no significant difference (*p* = 0.46, Student’s *t* test) in EDSS scores at nadir of disease of patients who recovered completely (mean EDSS 7.9) and patients that showed only partial recovery (mean EDSS 7.3).

## Discussion

LETM is a heterogeneous disorder with various causes including infectious, acquired autoimmune and connective tissue disorders resulting in different clinical features and outcome. In our study, we identified first clinical presentation of MOG-EM as the underlying cause of LETM in 11% of patients who displayed quite similar clinical, radiological and CSF phenotypes. When comparing our findings with two larger cohorts of patients with MOG-EM, the German NEMOS cohort and Australasian/New Zealand MOG study group, preceding infections prior to first attack of MOG-EM were reported in 11/50 (22%; NEMOS) and 28/59 (47%; AUS) patients. Taking into account all attacks, 15/37 (41%) patients in the NEMOS group reported at least one attack preceded by infection. First clinical attack was preceded by vaccinations in 2/50 (4%; NEMOS) and 2/59 (3%; AUS) patients [8, 16, 23]. Our cohort is in line with these findings (4/7: cases preceded by infection; 1/7: preceded by vaccination) pointing towards the importance of external triggers for immune system activation in about 50% of patients. Moreover, there has been a previous report in which MOG-IgG-positive myelitis has been associated with thyroid gland disease [16] while these findings should currently not be overemphasized due to the high prevalence of thyroid diseases.

Spinal cord MRI findings in the NEMOS cohort reported contrast enhancement in 19/28 (67%) patients [16]. Other reports also state a regular occurrence of gadolinium enhancement in both MOG- and aquaporin-4 positive LETM [26]. Interestingly, this is in contrast to our results (0/7 patients). It should be noted that previous investigations assessed spinal cord images irrespective of the time point during the disease course at which MRI images were taken and that our findings might, therefore, be characteristic for the specific subgroup of patients with MOG-EM presenting with LETM only as first clinical attack. Of note, 55.7% of the 62 patients with LETM showed gadolinium enhancement of lesions in spinal cord MRI. Lack of gadolinium enhancement, although not exclusive to MOG-EM, could, therefore, be a feature of MOG-EM with LETM as first presentation and should lead to testing of MOG-IgG in these patients. Only limited data are available for whether MOG-IgG antibody titers, which might primarily be involved in causing demyelinating lesions, are useful to monitor disease courses. In a recent study, titers > 1:2560 were only found in a short time window at acute attack, while other patients still had relatively low titers during acute attacks or high titers during remission [23]. In our study we used commercial fixed cell based assays to measure MOG antibodies. This is a possible limitation to our study as recent studies demonstrate that this assay has a lower sensitivity and specificity compared to live cell-based assays and could, therefore, lead to false positives in patients with low titers [27, 28]. Although some of our patients had relatively low titers, we see the diagnosis of MOG-EM confirmed in the typical clinical association [2]. Moreover, our cohort has been retested for MOG-IgG in remission and in 5/7 patients MOG-IgG could no longer be detected. All these patients presented with a monophasic disease course during our observation period and the only patient in our cohort with a second relapse stayed MOG-IgG positive. There have been previous reports about patients, especially with a monophasic disease course in which MOG-IgG disappeared over time. However, these studies mainly reported on children and juveniles and lacked long-term data or did not specifically address LETM as first presentation of MOG-EM [2, 7, 29–31]. Our findings emphasize that, also in adults, disappearance of MOG-IgG can hint towards a monophasic disease course. Re-testing of MOG-IgG positive patients after recovery from the first attack should, therefore, be considered to inform treatment decisions in MOG-EM patients.

Patients responded well to plasma exchange or intravenous immunoglobulin but not to corticosteroids during acute attack. This suggests a direct pathological role of the MOG-IgG antibody as has been discussed previously [1]. Previous reports described that corticosteroids were not always effective and were followed by partial or no recovery in 50% of treated attacks [16]. In none of our patients corticosteroids were followed by complete recovery, underlining a higher efficacy of plasma exchange treatment in patients with LETM as a first presentation of disease.

In our cohort, all patients were treated with long-term immunotherapy after first acute attack. Previous observational studies have demonstrated a beneficial effect of rituximab in reducing relapse rate in patients [8]. This is consistent with observations in our study, in which 6/7 patients were treated with rituximab and we observed a low relapse rate, though further investigation would be needed to confirm whether this correlation is also causative.

During our observation period, we observed a low relapse rate of 14% (one out of seven patients). This might be due to the efficacy of the immunosuppressive treatment but could also hint towards a monophasic disease course in MOG-IgG positive disease as discussed in previous studies [16, 32–34]. Relapses in MOG-EM seem to occur later than in NMOSD (only one attack after 12–24 months in MOG-EM: 41–70% versus NMOSD: 7–29%) [4, 5, 34]. Altogether, longer observation periods show a drastically higher percentage of patients with at least one second attack. In a longer follow-up study of 43 months, only 29% of patients had a monophasic disease course and after 8 years, this patient group went down to only 7% [26]. This underlines that a severe clinical onset of MOG-EM should rather justify a prolonged treatment period with immunomodulatory drugs as the second clinical attack can occur only after an interval of several years.

## Conclusion

In summary, we identified rapid and severe onset, previous infections, lack of gadolinium enhancement in spinal cord MRI and strong pleocytosis and negative oligoclonal bands in CSF analysis as common pathological findings in patients with LETM as first clinical presentation of MOG-EM. While none of these clinical and paraclinical markers is exclusive for MOG-EM, patients with aquaporin-4 negative LETM should be routinely tested for MOG-IgG as a marker for MOG-EM in these cases. Disappearance of MOG-seropositivity in remission is likely associated with a monophasic disease course.

## Availability of data and material

The datasets generated and analyzed during the current study are not publicly available due to data restrictions to keep the privacy of the patients, but are available from the corresponding author on reasonable request and with permission of the Regional Ethics Committee.

## Abbreviations

MOG-EM: MOG-IgG-associated encephalomyelitis
MS: Multiple sclerosis
NMOSD: Neuromyelitis optica spectrum disorder
LETM: Longitudinal extensive transverse myelitis
ADEM: Acute disseminated encephalomyelitits
ON: Optic neuritis
MRI: Magnetic resonance imaging
FLAIR: Fluid-attenuated inversion recovery
CSF: Cerebrospinal fluid
EDSS: Expanded disability status scale
VEP: Visual evoked potential

## References

[1] Reindl M, Waters P (2019). Myelin oligodendrocyte glycoprotein antibodies in neurological disease. Nat Rev Neurol.

[2] Jarius S, Paul F, Aktas O, Asgari N, Dale RC, de Seze J (2018). MOG encephalomyelitis: international recommendations on diagnosis and antibody testing. J Neuroinflamm.

[3] Weber MS, Derfuss T, Bruck W (2018). Anti-myelin oligodendrocyte glycoprotein antibody-associated central nervous system demyelination-a novel disease entity?. JAMA Neurol.

[4] Dos Passos GR, Oliveira LM, da Costa BK, Apostolos-Pereira SL, Callegaro D, Fujihara K (2018). MOG-IgG-associated optic neuritis, encephalitis, and myelitis: lessons learned from neuromyelitis optica spectrum disorder. Front Neurol.

[5] van Pelt ED, Wong YY, Ketelslegers IA, Hamann D, Hintzen RQ (2016). Neuromyelitis optica spectrum disorders: comparison of clinical and magnetic resonance imaging characteristics of AQP4-IgG versus MOG-IgG seropositive cases in the Netherlands. Eur J Neurol.

[6] McLaughlin KA, Chitnis T, Newcombe J, Franz B, Kennedy J, McArdel S (2009). Age-dependent B cell autoimmunity to a myelin surface antigen in pediatric multiple sclerosis. J Immunol.

[7] Probstel AK, Dornmair K, Bittner R, Sperl P, Jenne D, Magalhaes S (2011). Antibodies to MOG are transient in childhood acute disseminated encephalomyelitis. Neurology.

[8] Ramanathan S, Mohammad S, Tantsis E, Nguyen TK, Merheb V, Fung VSC (2018). Clinical course, therapeutic responses and outcomes in relapsing MOG antibody-associated demyelination. J Neurol Neurosurg Psychiatry.

[9] Hennes EM, Baumann M, Lechner C, Rostasy K (2018). MOG Spectrum Disorders and Role of MOG-Antibodies in Clinical Practice. Neuropediatrics.

[10] Cobo-Calvo A, Ruiz A, D'Indy H, Poulat AL, Carneiro M, Philippe N (2017). MOG antibody-related disorders: common features and uncommon presentations. J Neurol.

[11] Pandit L, Sato DK, Mustafa S, Takahashi T, D'Cunha A, Malli C (2016). Relapsing optic neuritis and isolated transverse myelitis are the predominant clinical phenotypes for patients with antibodies to myelin oligodendrocyte glycoprotein in India. Multiple Scler J Exp Transl Clin.

[12] Cobo-Calvo A, Ruiz A, Maillart E, Audoin B, Zephir H, Bourre B (2018). Clinical spectrum and prognostic value of CNS MOG autoimmunity in adults: The MOGADOR study. Neurology.

[13] Dubey D, Pittock SJ, Krecke KN, Morris PP, Sechi E, Zalewski NL (2019). Clinical, radiologic, and prognostic features of myelitis associated with myelin oligodendrocyte glycoprotein autoantibody. JAMA Neurol.

[14] Sechi E, Shosha E, Williams JP, Pittock SJ, Weinshenker BG, Keegan BM (2019). Aquaporin-4 and MOG autoantibody discovery in idiopathic transverse myelitis epidemiology. Neurology.

[15] Tantsis EM, Prelog K, Alper G, Benson L, Gorman M, Lim M (2019). Magnetic resonance imaging in enterovirus-71, myelin oligodendrocyte glycoprotein antibody, aquaporin-4 antibody, and multiple sclerosis-associated myelitis in children. Dev Med Child Neurol.

[16] Jarius S, Ruprecht K, Kleiter I, Borisow N, Asgari N, Pitarokoili K et al (2016) MOG-IgG in NMO and related disorders: a multicenter study of 50 patients. Part 2: Epidemiology, clinical presentation, radiological and laboratory features, treatment responses, and long-term outcome. J Neuroinflamm 13(1):280 10.1186/s12974-016-0718-0 PMC5086042 27793206

[17] Trebst C, Raab P, Voss EV, Rommer P, Abu-Mugheisib M, Zettl UK (2011). Longitudinal extensive transverse myelitis–it's not all neuromyelitis optica. Nat Rev Neurol.

[18] Thompson AJ, Banwell BL, Barkhof F, Carroll WM, Coetzee T, Comi G (2018). Diagnosis of multiple sclerosis: 2017 revisions of the McDonald criteria. Lancet Neurol.

[19] Flanagan EP, Kaufmann TJ, Krecke KN, Aksamit AJ, Pittock SJ, Keegan BM (2016). Discriminating long myelitis of neuromyelitis optica from sarcoidosis. Ann Neurol.

[20] Asgari N, Flanagan EP, Fujihara K, Kim HJ, Skejoe HP, Wuerfel J et al (2017) Disruption of the leptomeningeal blood barrier in neuromyelitis optica spectrum disorder. Neurol Neuroimmunol Neuroinflamm 4(4):e343 10.1212/NXI.0000000000000343 PMC5400808 28451627

[21] Wingerchuk DM, Banwell B, Bennett JL, Cabre P, Carroll W, Chitnis T (2015). International consensus diagnostic criteria for neuromyelitis optica spectrum disorders. Neurology.

[22] Wingerchuk DM, Lennon VA, Pittock SJ, Lucchinetti CF, Weinshenker BG (2006). Revised diagnostic criteria for neuromyelitis optica. Neurology.

[23] Jarius S, Ruprecht K, Kleiter I, Borisow N, Asgari N, Pitarokoili K et al (2016) MOG-IgG in NMO and related disorders: a multicenter study of 50 patients. Part 1: Frequency, syndrome specificity, influence of disease activity, long-term course, association with AQP4-IgG, and origin. J Neuroinflamm 13(1):279 10.1186/s12974-016-0717-1 PMC5084340 27788675

[24] Franciotta D, Columba-Cabezas S, Andreoni L, Ravaglia S, Jarius S, Romagnolo S (2008). Oligoclonal IgG band patterns in inflammatory demyelinating human and mouse diseases. J Neuroimmunol.

[25] Jarius S, Paul F, Franciotta D, Ruprecht K, Ringelstein M, Bergamaschi R (2011). Cerebrospinal fluid findings in aquaporin-4 antibody positive neuromyelitis optica: results from 211 lumbar punctures. J Neurol Sci.

[26] Narayan R, Simpson A, Fritsche K, Salama S, Pardo S, Mealy M (2018). MOG antibody disease: A review of MOG antibody seropositive neuromyelitis optica spectrum disorder. Multiple Scler Relat Disord.

[27] Tea F, Lopez JA, Ramanathan S, Merheb V, Lee FXZ, Zou A (2019). Characterization of the human myelin oligodendrocyte glycoprotein antibody response in demyelination. Acta Neuropathol Commun.

[28] Waters P, Vincent A (2019). Myelin oligodendrocyte glycoprotein CSF testing needs testing. Neurology.

[29] Di Pauli F, Mader S, Rostasy K, Schanda K, Bajer-Kornek B, Ehling R (2011). Temporal dynamics of anti-MOG antibodies in CNS demyelinating diseases. Clin Immunol (Orlando, Fla).

[30] Hoftberger R, Sepulveda M, Armangue T, Blanco Y, Rostasy K, Calvo AC (2015). Antibodies to MOG and AQP4 in adults with neuromyelitis optica and suspected limited forms of the disease. Multiple Scler (Houndmills, Basingstoke, England).

[31] Oliveira LM, Apostolos-Pereira SL, Pitombeira MS, Bruel Torretta PH, Callegaro D, Sato DK (2019). Persistent MOG-IgG positivity is a predictor of recurrence in MOG-IgG-associated optic neuritis, encephalitis and myelitis. Multiple Scler (Houndmills, Basingstoke, England).

[32] Kitley J, Woodhall M, Waters P, Leite MI, Devenney E, Craig J (2012). Myelin-oligodendrocyte glycoprotein antibodies in adults with a neuromyelitis optica phenotype. Neurology.

[33] Kitley J, Waters P, Woodhall M, Leite MI, Murchison A, George J (2014). Neuromyelitis optica spectrum disorders with aquaporin-4 and myelin-oligodendrocyte glycoprotein antibodies: a comparative study. JAMA Neurol.

[34] Sato DK, Callegaro D, Lana-Peixoto MA, Waters PJ, de Haidar Jorge FM, Takahashi T (2014). Distinction between MOG antibody-positive and AQP4 antibody-positive NMO spectrum disorders. Neurology.

